# Comparative Host Response of 2 Human Acellular Dermal Matrices in a Primate Implant Model

**Published:** 2014-01-31

**Authors:** Maryellen Sandor, Devinder Singh, Ronald P. Silverman, Hui Xu, Patrick G. De Deyne

**Affiliations:** ^a^LifeCell Corporation, Bridgewater, NJ; ^b^University of Maryland School of Medicine, Division of Plastic Surgery, Baltimore, MD

**Keywords:** acellular dermal matrix, AlloDerm, animals, breast implants, foreign-body reaction, tissue expanders

## Abstract

**Objective:** We examined the differences in capsule formation between 2 commercially available human acellular dermal matrices in a nonhuman primate model. **Methods:** Primates were implanted dorsally with a subcutaneously placed tissue expander and randomized into 3 groups, receiving skin coverage only, coverage with non-irradiated freeze-dried human acellular dermal matrix, or coverage with gamma-irradiated human acellular dermal matrix. After 9 weeks, soft tissue around the tissue expander was excised and evaluated qualitatively and quantitatively to assess extent of inflammation (CD68 antibodies and interleukin-6 levels), degradation and fibrosis (matrix metalloproteinase-1 and procollagen-1 staining), and mechanical (tensile) strength. **Results:** Histological evaluation of tissue around the tissue expander indicated differences in host response, suggesting capsule presence in the gamma-irradiated matrix group but not the freeze-dried matrix group. The extent of local inflammation was much higher in the gamma-irradiated matrix group which demonstrated mean (standard deviation) localized interleukin-6 concentration of 67.3 (53.6) vs 16.3 (6.7) pg/mg protein in the non-irradiated matrix group. There was robust degradation and fibrotic response in the gamma-irradiated matrix group versus the freeze-dried matrix group. Mechanical testing indicated mean (standard deviation) ultimate tensile strength of 12.0 (7.1) N in the gamma-irradiated matrix group versus 99.3 (48.8) N in the freeze-dried matrix group. **Conclusions:** Enclosure of a tissue expander with human acellular dermal matrix untreated by gamma irradiation led to minimal inflammation and minimal evidence of fibrosis/capsule around the tissue expander compared with robust capsule formation around the tissue expander that was covered by a gamma-irradiated human acellular dermal matrix.

Implant-based breast reconstruction following mastectomy is a widely used alternative to autologous reconstruction techniques. In many cases, a 2-stage tissue expander (TE)/implant exchange procedure is employed. As with any implanted device, a foreign-body reaction may lead to the formation of a thin layer of scar tissue, or capsule, surrounding the TE or implant, which may be associated with capsular contracture.^1^^,^^2^ Capsular contracture is seen in 10% to 15% of breasts following reconstruction,^3^ and severe contracture may lead to deformity and the need for reoperation.^4^^-^6 Moreover, the occurrence of capsular contracture seems to be associated with radiation therapy.^7^

The current prevailing hypotheses for the cause of capsular contracture are an association with bacterial infection^8^ or the result of a chronic inflammatory cellular environment around the implant.^3^^,^^9^ Following implantation of a synthetic material, inflammatory cells migrate to the implant site, and levels of associated inflammatory cytokines and tissue remodeling factors including matrix metalloproteinases (MMPs) and growth factors become elevated.^10^^,^^11^ Subsequent migration of fibroblasts and myofibroblasts to the site is accompanied by production of procollagen-1 and alpha-smooth muscle cell actin (α-SMA) followed by development of dense, fibrotic tissue.^2^^,^^12^^,^^13^ The presence of many of these biochemical markers is evident in the normal wound healing and remodeling process (CD-68, matrix metalloproteinase-1 [MMP-1], tissue inhibitor of metalloproteinase-1 [TIMP-1]). However, these events, when prolonged in excess and coupled with resulting excessive α-SMA and procollagens presence are also presumed to be related to the formation of capsular contracture in breast reconstruction, which is currently described as a synovia-like metaplasial cell layer that forms at the implant-capsule interface.^12^^-^14

The clinical usage and efficacy of acellular dermal matrices (ADMs) have been analyzed thoroughly, and recent meta-analyses also support their utilization in breast reconstruciton.^15^^,^^16^ Reports on in vitro comparative testing of ADMs have also been published,^17^^,^^18^ but few studies have focused on the characterization of the ADM in the presence of a TE,^9^^,^^19^ especially in primates.^20^ A more detailed characterization of the ADM-induced inflammatory, degradation, and fibrotic host responses is in order in the field of regenerative medicine, because it has recently been observed that macrophages can change their phenotype and foster constructive remodeling.^21^ A prior study suggests that human ADMs (HADMs) used in conjunction with a TE may be useful for reducing capsule formation and fibrosis in the breast implant setting.^20^

The objective of the current study was to more thoroughly characterize the local tissue response in the previously reported nonhuman primate model^20^ (inflammation, degradation/fibrosis, and mechanics) after partially enclosing a TE with commercially available human dermal-derived grafts that were either processed aseptically and freeze-dried or sterilized using gamma irradiation. Previous studies with ADMs have indicated a regenerative response to those processed without using caustic reagents or gamma-irradiation^22^^,^^23^ and an inflammation/degradation response to those with an altered collagen matrix.^24^ We hypothesized that there would be differences in the extent of regenerative response with respect to inflammatory response, degradation, fibrosis, and mechanical properties of the soft tissue explants in a primate model when differently processed HADMs were used with a TE.

## METHODS

### Study overview

The experimental protocol was approved by the Institutional Animal Care and Use Committee of the Behavioral Sciences Foundation, St Kitts, Eastern Caribbean. Behavioral Sciences Foundation is accredited with the Canadian Council for Animal Care.

Nine adult male Caribbean vervets (Chlorocebus aethiops) (3–6 kg) were included in the study. Animals were randomly assigned to 1 of 3 treatment groups to receive a subcutaneous TE implant either on the left or right side of the dorsum. Animals were randomized to receive (1) TE only and TE with an overlay of aseptically processed HADM (TE + AD, with AD being AlloDerm Regenerative Tissue Matrix, LifeCell Corporation, Branchburg, New Jersey); (2) TE only and TE with an overlay of a gamma-irradiated HADM (TE + AM, with AM being AlloMax, Bard-Davol, Warwick, Rhode Island); or (3) TE + AD and TE + AM (Table 1). At 9 weeks following implantation, animals were killed and soft tissue around the TE was harvested. The TE-only group was used as a control (surgery, TE implant, skin flap coverage, no HADM).

### TE implantation

Animals were fasted for 24 hours prior to the procedure and anesthetized by intramuscular injection of ketamine (10 mg/kg) and xylazine (1.0 mg/kg). Each animal was then placed in ventral recumbency and its upper back shaved and aseptically prepared for surgery. A single 125-mg dose of cefazolin was given intramuscularly before incision. Two horizontal curved incisions (≥6 cm) were made through the skin and subcutaneous tissues on either side of the back below the shoulder blades. Dorsal, rather than ventral, implantation was performed to prevent host postoperative tampering. A subcutaneous pocket was created above the dorsal musculature and deep fascia on either side of the spine to accommodate a 30-mL, 3-cm diameter circular, smooth silicone shell TE (PMT Corp, Chanhassen, Minnesota). For TE-only treatment, a TE was inserted into the subcutaneous pocket and filled with 25-mL sterile saline solution. The port and tubing were ligated and removed, and the TE anchored to the fascia with 2-0 polypropylene sutures around the port remnant. For the TE + AD and TE + AM groups, a 6 × 6 cm^2^ dermal graft sheet (1.04-2.28 mm thick for AD; 0.8-1.8 mm thick for AM) was placed into the subcutaneous pocket and sutured down in a circumferential pattern to the underlying dorsal musculature. A TE was inserted above the muscle and positioned such that it was completely covered by dermal graft. The subcutaneous layer was then closed with polydioxanone sutures in a continuous subcuticular pattern, and skin was closed using nonabsorbable nylon sutures in an interrupted pattern. Animals received a 3-day course of cefazolin (125 mg bid, intramuscularly [IM]) with Banamine (Schering-Plough Animal Health, Kenilworth, New Jersey) 5 mg/kg given as needed. Animals were given flunixin meglumine (2.0–5.0 mg/kg, IM) or buprenorphine (0.01 mg/kg subcutaneously or IM) immediately after surgery, with additional analgesic permitted twice daily for 3 days or longer for persistent pain. All animals underwent biweekly physical examinations to monitor for complications, particularly at the surgical site.

### Tissue harvesting

Animals were euthanized by intravenous injection of pentobarbital 9 weeks postimplantation. TE and surrounding tissues were removed en bloc, inclusive of the implanted dermal graft material, host tissues above the implanted TE, and the thoracic muscle below the TE. The entire explanted surgical pocket was then carefully divided along a sagittal plane into thirds, with the lateral one-third flash frozen in dry ice for later immunochemical analysis and the remaining two-thirds divided for fixation in 10% formalin (lateral segment) pending histologic and immunohistochemical analysis or immediate biomechanical testing (central unfixed segment).

### Tissue assessments

Paraffin-embedded tissue samples were sectioned and underwent routine hematoxylin and eosin (H&E) staining and Verhoeff-Van Gieson (VVG) staining for elastin. For immunohistochemical labeling, tissue sections were deparaffinized and rehydrated, and proteinase K applied for antigen retrieval. Monoclonal antibodies to α-SMA, CD68, MMP-1, TIMP-1, and procollagen-1 were applied using the appropriate dilutions (Table 2). Detection was achieved using an appropriate secondary anti-immunoglobulin G (Table 2) conjugated with horseradish peroxidase and labeling was visualized with diaminobenzidine. Sections were evaluated for localization and intensity of staining for each immunohistochemical marker, scored by 2 independent histopathologists who were blinded to the nature of the individual samples, and the scores for each biopsy averaged. Immunohistochemical detection of the selected markers was used to determine the presence of inflammation (CD68), degradation (MMP-1/TIMP-1), characterization of capsule formation (α-SMA), and fibrosis (procollagen-1). H&E- and VVG-stained slides were evaluated for the presence of capsule and graft resorption as indicated by heterogeneous distribution of dermal elastin, respectively. Biopsies were scored for these factors as either present or absent. Relative presence and staining for each individual immunohistochemical marker was compared to the appropriate positive control, with a score of “−” indicating 0% staining, “+/−” indicating 5% to 20% of positive control, “+” indicating 20% to 40% of positive control, “++” indicating 40% to 60% of positive control, “+++” indicating 60% to 80% of positive control, and “++++” indicating 80% to 100% or equivalent to positive control as indicated in Table 2.

For biochemical analysis, flash-frozen samples were extracted and analyzed for inflammatory cytokines using a multiplex immunoassay array for biochemical markers of inflammation and fibrosis, including interleukins (ILs) 1, 2, 4, 6, 8, and 10, interferon (INF)-γ, regulated upon activation normal T cell expressed and secreted (RANTES), tumor necrosis factor (TNF)-α, and vascular endothelial growth factor (Bio-Rad, Hercules, California, cat M50-000007A). Briefly, frozen samples were pulverized in a tissue cryohomogenizer, followed by treatment with cell lysis kit solution (Bio-Rad, cat 171-304011) according to manufacturer's instructions. Samples were shaken for 15 minutes followed by centrifugation and the supernatant collected. For total protein content, an aliquot of supernatant was mixed with Bradford protein assay reagent (Bio-Rad, cat 500-0006), added to a 96-well plate and read at 595 nm. Additional supernatant aliquots were added to a 96-well plate incubated and rinsed according to manufacturer's instructions. On the basis of the array results showing consistently detectable concentrations of IL-6, INF-γ, and TNF-α in tissue explants, we validated the results using a 3-plex enzyme-linked immunosorbent assay (BioRad).

Uniaxial tensile loading was conducted on freshly dissected dermal graft samples for TE + AD and TE + AM groups. Briefly, a 1 × 3 cm^2^ strip was placed in pneumatic side action grips with rubber faces and tested at a controlled strain rate of 1.65 per minute until failure (Instron 5860; Norwood, Massachusetts). Outcome measures included ultimate strength and elastic modulus. Data were processed using Instron Bluehill software and Microsoft Excel.

### Data analysis

All implant sites were evaluated, with the exception of those where the TE extruded prior to scheduled explant date. Density and qualitative features of routine histologic staining and immunolabeling were described and scored independently by 2 independent histologists who were blinded to the nature of the individual samples, taking into account the entirety of 3 biopsy sections each, when viewed at 40×, 100×, and 200× magnifications. Scores were averaged to obtain the overall score for each sample, and the qualitative scores were compared across TE + AM and TE + AD groups using a Mann-Whitney test. For immunochemical evaluation, cytokine concentration for each sample was normalized to the total protein concentration and results reported in picogram analyte per milligram total protein. Immunochemical testing results from all evaluable specimens dissected from the overlying subcutaneous tissues were compared between the treatment groups using a 2-way analysis of variance model, following transformation to the normal distribution for biochemical results, with group defined as a factor; significance was defined as *P* ≤ .05. For mechanical testing results, a student *t* test was used to determine significance between individual groups.

## RESULTS

### Postoperative recovery

Two of the animals experienced extrusion of TEs before killing (1 animal [07657] with implant extruded from a TE + AD site at week 4.5, while the other side [TE + AM] was intact and 1 animal [07655] with both TEs extruded [TE-only and TE + AD] at week 1.5). The taut nature of the skin and observed necrotic tissue overlying these 3 extruded TEs early in the experiment indicated pressure necrosis due to overinflation of these particular TEs in correspondingly tight subcutaneous pockets. These surgical sites were not included in the analysis. No other surgical sites were observed to have pressure necrosis-induced complications.

### Morphology: Histology and immunohistochemistry of implanted HADM

**Figure 1:**
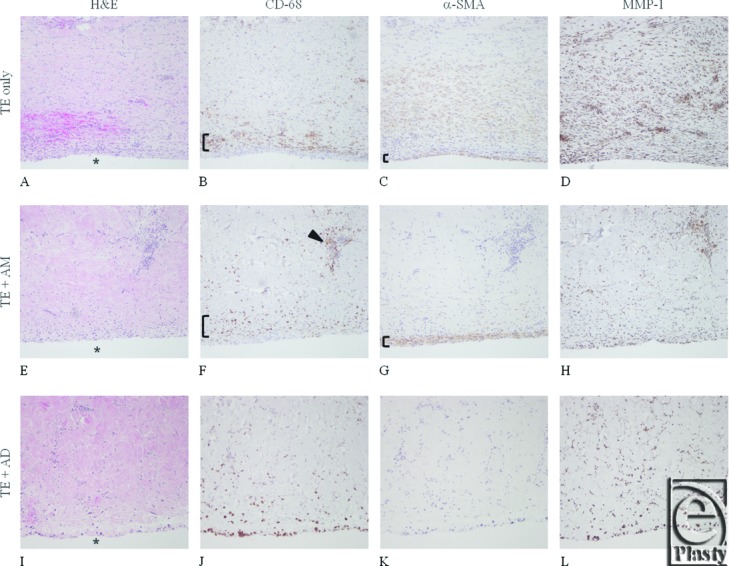
Representative histology and immunohistochemical staining using serial sections of tissue overlying the implanted tissue expander (TE) interface (indicated by *) for the TE-only (A-D), AlloMax (AM; E-H), and AlloDerm (AD; I-L) groups. Tissues are stained/labeled for hematoxylin and eosin (A, E, I); CD68 epitope for macrophages (B, F, J); α-smooth muscle cell actin (SMA) for myofibroblasts (C, G, K); and matrix metalloproteinase-1 (MMP-1) for matrix collagenase (D, H, I). In the TE-only group, a rim of macrophages can be detected ([, panel B) and presence of myofibroblasts ([, panel C). Tissue around the AM-covered TE had more modest macrophage presence ([, panel F); however, clusters were observed (indicated by arrowhead in F) and myofibroblast staining was strong ([, panel G). The lowest signal for macrophages and myofibroblast was noted in tissues from AD-covered TE. Excessive MMP-1 staining (D, H, L) is suggestive of accelerated ECM degradation in TE-only and clustered activity in AM (not observed in tissue from AD-covered TE).

H&E and VVG staining confirmed the presence of both HADM types in the areas overlying the TE. There were, however, qualitative differences between the 2 groups. The soft tissue in the TE + AD group showed little to no sign of graft resorption, illustrated by an even distribution of elastin. Histology indicated the presence of fibroblasts and neovascularization within the AD graft, infiltrating from the matrix-dermis interface and not yet contacting the matrix-TE interface below, indicated by an asterisk (*) in Figure 1I. The immunohistochemistry of the soft tissue in the TE + AD group showed moderate presence of MMP-1 (Fig 1L, Table 3), with relatively little evidence of TIMP-1 (Table 3). Few α-SMA–labeled myofibroblasts were seen (Fig 1K), with only 1 of the 4 TE + AD sites exhibiting a single layer of α-SMA–positive myofibroblasts at the TE-matrix interface (Table 3). Procollagen-1 labeling was observed within the matrix and at the periphery (Table 3). There were few CD68-positive macrophages observed (Fig 1J, Table 3). On the contrary, the immunohistochemical profile of the soft tissue in the TE + AM group was different with regard to intensity and distribution. In 5 of 6 TE + AM implants evaluated, staining showed the presence of HADM with nonuniform thickness (Fig 1E), marked by thinned areas and a dense elastin fiber concentration, uncharacteristic of dermal tissue and suggesting matrix resorption. Areas of robust MMP-1 staining (Fig 1H), coupled with moderate TIMP-1 staining provided strong evidence for a different staining pattern compared to the AD group (Table 3). The α-SMA–positive myofibroblasts at the TE-matrix interface (Fig 1G, Table 3) suggested the presence of robust capsule formation in the TE + AM group. Procollagen-1 staining was negligible to moderate. Overlying tissue from TE + AM capsules demonstrated areas of significant macrophage presence (Fig 1F, Table 3) exhibiting a synovial-like metaplasia at the TE-matrix interface. Qualitative scores were compared across TE + AM and TE + AD groups using a Mann-Whitney test; *P* values were as follows: α-SMA, *P* = .04; CD68, *P* = .15; MMP-1, *P* = .08; TIMP-1, *P* = .12); procollagen-1, *P* = .15.

#### TE-Only

All 5 TE-only sites exhibited α-SMA–positive capsule formation at the dermis-TE interface (Fig 1C, Table 3), with 2 sites also demonstrating a significant presence of CD68-labeled macrophages (Fig 1B). In 2 TE-only sites, the overlying host skin was marked by the presence of sparse collagen, coinciding with moderate to significant expression of MMP-1 (Fig 1D).

### Immunoassay

Concentrations of IL-1, IL-2, IL-4, IL-8, IL-10, RANTES, and vascular endothelial growth factor in all 3 groups were not detectable within the linear range of the assay. Measurable results were obtained for TNF-α, INF-γ, and IL-6 using the same array. Repeat analysis was performed to validate the array data and a separate 3-plex array for TNF-α, INF-γ, and IL-6 revealed that neither TNF-α nor INF-γ were significantly different among the 3 groups for samples collected from tissues overlying the TEs (Table 4). Analysis of variance revealed statistical significance across the treatment groups for IL-6 results (*P* = .006). Significantly lower levels of IL-6 were detected in tissues from the TE + AD group as compared to both the TE + AM (*P* = .004) and TE-only groups (*P* = .02) as determined by *t* test between individual groups. Interleukin-6 tissue concentrations were not significantly different between TE + AM and TE-only groups.

### Mechanical strength testing

Table 5 shows the mechanical properties of HADMs, both out-of-package and following implantation for 9 weeks. While AM out-of-package tensile strength (6.19 ± 0.79 MPa) was significantly less than that of AD (10.93 ± 4.61 MPa) (*P* < .01), both HADMs exhibited a change in mechanical properties following implantation, with AD retaining ˜55% mechanical strength in vivo (6.00 ± 2.55 MPa) and AM retaining ˜12% (0.73 ± 0.46 MPa) (*P* < .001), in line with histology findings indicating significant resorption of AM. The elastic modulus of AD was significantly higher than AM, both out-of-package (48.85 ± 24.43 vs 17.42 ± 2.49 MPa, respectively; *P* < .001) and following implant (20.48 ± 5.80 vs 3.51 ± 3.43 MPa, respectively; *P* < .001).

## DISCUSSION

Several large published series of patients undergoing breast reconstruction utilizing HADM have reported very low rates of capsular contracture.^25^ In addition, a nonhuman primate study reported the absence of capsule around implants where HADM was placed.^20^ The absence of a capsule at the site of HADM placement has been observed clinically by the authors at the time of implant exchange and was recently confirmed histologically in a clinical study where biopsies were obtained at the time of implant exchange.^26^ The purpose of this study was to determine whether different processing of the HADM has an impact on the formation of capsule in this established primate model.

Our data suggest that HADMs that are used in breast reconstruction lead to a difference in the host response. More specifically, HADM that is processed with caustic reagents and gamma irradiation (AM) led to a local tissue response that was more similar to the soft tissue around the TE-only control group. The AM-TE interface was characterized by a localized substantial presence of CD68-positive macrophages adjacent to a continuous layer of α-SMA–positive myofibroblasts, suggesting robust inflammatory and fibrotic processes potentially associated with synovial metaplasia. The staining pattern of the non–gamma-irradiated HADM (AD) at the TE interface was quite different in that few CD68-positive macrophages were present, typical of normal wound healing, with minimal to no presence of α-SMA–positive myofibroblasts. When these morphological data were analyzed in conjunction with a quantitative biochemical test, the contrast between the 2 HADMs was even more evident, with a fourfold increase in IL-6 concentration and a nearly 90% drop in out-of-package mechanical strength of the gamma-irradiated HADM over the study duration.

Detailed comparisons of ADMs have been performed,^17^^,^^27^ and it is interesting to note that collagenase treatment of a gamma-irradiated scaffold led to a rapid loss in material properties (elastic modulus) compared to a non–gamma-irradiated scaffold, despite similar out-of-package mechanical properties. It is well recognized that gamma irradiation of human collagen matrices leads to an altered collagen morphology and decreased mechanical properties^28^ as well as altered hydration properties.^29^ A recent in vitro study also demonstrated significant differences in the biochemical composition, thermal properties, and biomechanics of gamma-irradiated human dermal matrices in comparison to matrices treated with electron-beam irradiation with the addition of antioxidative agents, or nonirradiated matrices. The e-beam and nonirradiated tissues retained properties similar to native dermis,^30^ which may be important for regeneration.^22^^-^24 Measuring the strength of the soft tissue around the TE following in vivo implantation may be an important variable, as loss of HADM thickness and decrease in mechanical properties with degradation may have led to capsule formation in the case of AM, while clinical data have indicated an increase in stiffness of capsule with contracture severity.^31^ It has been shown in the literature that gamma irradiation of collagen-based materials without the addition of antioxidative agents leads to collagen fragmentation and generation of free radicals, and that this effect is a function of both the dosage and the state (wet/dry, ambient/frozen) of the material at the time of treatment.^28^ In this study, the immunohistochemistry showed that the AM group also had robust MMP-1 staining focused around clusters of macrophages, suggesting the association of inflammation with degradation. The soft tissue around the TE + AD samples showed a different and less intense staining pattern, without a band of myofibroblasts, unlike the gamma-irradiated material (TE + AM).

Our data complement the work from previous studies reporting on the characterization of biological scaffold–synthetic material interfaces,^32^^,^^33^ especially when AD is tested in conjunction with radiation therapy, a condition that predisposes patients to develop capsular contraction. Human acellular dermal matrix is thought to decrease radiation-induced inflammation and pseudoepithelium formation.^9^ The authors note that failure to test HADMs in the presence of irradiation is one of the possible limitations of the current animal study. Additional studies from the literature,^34^ which are not complimentary, focus solely on the in situ assessment of vascularization, rather than capsule formation. Another explanation for these differences may be the variation in animal models across studies.

Regenerative medicine focuses on replacement of dysfunctional organs or tissues with anatomically and physiologically functional tissues. Hence, it is important to assess the qualitative and quantitative features of implanted tissues. Studying the host response to ADMs by assessing markers for inflammation (CD68), fibrosis (α-SMA), and degradation (MMP-1) is an appropriate strategy and has been used previously when evaluating synthetic materials.^35^^,^^36^ Of the several cytokines that we evaluated in this study, only IL-6 was consistently elevated. We expected a broader cytokine footprint, such as that found in hypertrophic scarring, which has been reported to show increases in IL-1 and TNF-α in addition to IL-6.^37^ Nevertheless, we observed that HADM, when treated by gamma irradiation, leads to an inflammatory, fibrotic response that may be associated with synovial metaplasia, a feature of capsular contracture.

It should be noted that this study did not directly study capsular contracture, but rather the presence or absence of capsule at the 9-week time point. We hypothesize that the absence or delayed formation of capsule in that segment of a reconstructed breast pocket containing AD may contribute to a reduction in the rate of capsular contracture, and we believe longer-term study of this phenomenon is warranted.

## Figures and Tables

**Table 1:** Bilateral placement of TEs in a nonhuman primate model, with or without coverage by HADM

Animal ID	Placement	Implant condition
02703	Left	TE-only
	Right	TE + AM
N993	Left	TE-only
	Right	TE + AM
04099	Left	TE-only
	Right	TE + AM
01659	Left	TE-only
	Right	TE + AD
08247	Left	TE-only
	Right	TE + AD
07655	Left	TE-only
	Right	TE + AD
07656	Left	TE + AM
	Right	TE + AD
07654	Left	TE + AM
	Right	TE + AD
07657	Left	TE + AM
	Right	TE + AD

Treatment groups included tissue expander (TE)-only, TE + non–gamma-irradiated human acellular dermal matrix (HADM) (AlloDerm; AD), and TE + gamma-irradiated HADM (AlloMax; AM).

**Table 2:** Antibodies used for immunohistochemical detection

		Primary Antibody			Secondary Antibody		
Stain	Positive control	Species	Dilution	Supplier	Species	Dilution	Supplier
a-SMA	Monkey aorta	Mouse anti-human	1:60	Sigma A5691	Goat anti-mouse	1:300	Biorad 170-6516
CD-68	Monkey lymph node	Mouse anti-human	Pre-dilute	Zymed/Invitrogen 08-0125	Goat anti-mouse	1:300	Biorad 170-6516
MMP-1	Human ovarian tumor	Rabbit	1:600	Fitzgerald 10R-M112a	Rabbit polyclonal	Undiluted	Thermo Shandon TL-060-HL
TIMP-1	Human prostate tumor	Mouse	1:100	Fitzgerald 10R-M112b	Rabbit polyclonal	Undiluted	Thermo Shandon TL-060-HL
Procollagen -1	Human skin wound	Rat	1:600	Abcam ab64409	Goat anti-rat	Undiluted	Biocare GHP516 G

α-SMA indicates alpha-smooth muscle cell actin; CD, clusters of differentiation; MMP-1, matrix metalloproteinase-1; TIMP-1, tissue inhibitor of metalloproteinase-1.

**Table 3:** Histology/immunohistochemistry observations from tissues overlying the tissue expander for evaluable surgical sites

Animal/Implant ID	Resorption* (H&E/VVG)	Capsule Formation† (H&E)	α-SMA	CD68	MMP-1	TIMP-1	Procollagen-1
TE + AD							
08247R	Absent	Absent	−	+/−	+/−	−	−
01659R	Absent	Absent	−	+	++	−	−
07656R	Absent	Absent	−	+	++	+	++
07654R	Absent	Absent	+/−‡	+	++	+/−	+/−
TE + AM							
N993R	Present	Present	++	+/−	+++	++	++
02703R	Present	Absent	−	+++	++	+	+
04099R	Present	Present	+	+++	+++	+/−	+/−
07656L	Absent	Present	++	++	++	+	++
07657L	Present	Present	++	+	+++	+	++
07654L	Present	Present	+	++	+	+/−	+
TE-only							
08247L	N/A	Present	++	+	+++	+	+
01659L	N/A	Present	++	++	++	++	+
N993L	N/A	Present	++	+	+++	++	+
02703L	N/A	Present	++	+++	+++	+	+/−
04099L	N/A	Present	++	+++	++	+	+

*Resorption defined by loss of HADM thickness, uniformity of thickness (H&E), and uneven distribution of elastin (VVG).

†Capsule formation defined by multiple layers of collagen comprised linearly arranged fibroblast-like cells at the matrix-TE interface.

‡A single layer of a-SMA myofibroblasts was observed histologically at the matrix/TE interface.

α-SMA indicates alpha-smooth muscle actin; AD, AlloDerm; AM, AlloMax; CD, cluster of differentiation; H&E, hematoxylin and eosin; HADM, human acellular dermal matrix; MMP-1, matrix metalloproteinase-1; TE, tissue expander; TIMP-1, tissue inhibitor of metalloproteinase-1; VVG, Verhoeff-Van Gieson.

**Table 4:** In situ cytokine profile

	pg Cytokine/mg Total protein		
Implant group	IL-6	INF-γ	TNF-α
TE only	194.49 ± 288.20	25.81 ± 16.33	20.69 ± 5.78
TE + AD	16.33 ± 6.68	26.02 ± 10.69	18.29 ± 2.47
TE + AM	67.33 ± 53.59	16.98 ± 9.92	16.64 ± 2.63

AD indicates AlloDerm; AM, AlloMax; INF-γ, interferon-γ; IL-6, interleukin 6; TE, tissue expander; TNF-α, tumor necrosis factor-α.

**Table 5:** Biomechanical properties of HADMs following 9 weeks subcutaneous implantation enclosing a synthetic tissue expander

	Out-of-package properties, mean (SD)		Postimplantation properties, mean (SD)	
Material	Tensile strength maximum stress, MPa	Elastic modulus, MPa	Tensile strength maximum stress, MPa	Elastic modulus, MPa
AD	10.93 (4.61)	48.85 (24.43)	6.00 (2.55)	20.48 (5.80)
AM	6.19 (0.79)	17.42 (2.49)	0.73 (0.46)	3.51 (3.43)

AD indicates AlloDerm; AM, AlloMax; HADMs, human acellular dermal matrices.
